# Mitochondrial Medicine: A Promising Therapeutic Option Against Various Neurodegenerative Disorders

**DOI:** 10.2174/1570159X20666220830112408

**Published:** 2023-04-12

**Authors:** Mohannad A. Almikhlafi, Mohammed M. Karami, Ankit Jana, Thamer M. Alqurashi, Mohammed Majrashi, Badrah S. Alghamdi, Ghulam Md. Ashraf

**Affiliations:** 1 Department of Pharmacology and Toxicology, College of Pharmacy, Taibah University, Madinah, Saudi Arabia;; 2 Department of Physiology, Neuroscience Unit, Faculty of Medicine, King Abdulaziz University, Jeddah, Saudi Arabia;; 3 School of Biotechnology, Kalinga Institute of Industrial Technology (KIIT) Deemed to be University, Campus-11, Patia, Bhubaneswar, Odisha, 751024, India;; 4 Department of Pharmacology, Faculty of Medicine, Rabigh, King Abdulaziz University, Jeddah, Saudi Arabia;; 5 Department of Pharmacology, Faculty of Medicine, University of Jeddah, Jeddah, Saudi Arabia;; 6 Department of Physiology, Faculty of Medicine, King Abdulaziz University, Jeddah, Saudi Arabia;; 7 Pre-Clinical Research Unit, King Fahd Medical Research Center, King Abdulaziz University, Jeddah 21589, Saudi Arabia;; 8 The Neuroscience Research Unit, Faculty of Medicine, King Abdulaziz University, Jeddah, Saudi Arabia;; 9 Department of Medical Laboratory Sciences, College of Health Sciences, University of Sharjah, University City, Sharjah 27272, United Arab Emirates

**Keywords:** Alzheimer’s disease, amyotrophic lateral sclerosis, gene therapy, Huntington’s disease, mitochondrial dysfunction, Parkinson’s disease

## Abstract

Abnormal mitochondrial morphology and metabolic dysfunction have been observed in many neurodegenerative disorders (NDDs). Mitochondrial dysfunction can be caused by aberrant mitochondrial DNA, mutant nuclear proteins that interact with mitochondria directly or indirectly, or for unknown reasons. Since mitochondria play a significant role in neurodegeneration, mitochondria-targeted therapies represent a prosperous direction for the development of novel drug compounds that can be used to treat NDDs. This review gives a brief description of how mitochondrial abnormalities lead to various NDDs such as Alzheimer’s disease, Parkinson’s disease, Huntington’s disease, and amyotrophic lateral sclerosis. We further explore the promising therapeutic effectiveness of mitochondria-directed antioxidants, MitoQ, MitoVitE, MitoPBN, and dimebon. We have also discussed the possibility of mitochondrial gene therapy as a therapeutic option for these NDDs.

## 1 INTRODUCTION

The brain, despite accounting for just a small portion of our total body weight, is the greatest source of power, responsible for roughly 20% of total oxygen metabolism. Out of this, neurons are reported to utilize between 75-80% [1]. This energy is mostly used at the synapse, with a major percentage going towards restoring the depolarized neuronal membrane potentials. This high energy demand is constant, and even short durations of oxygen or glucose deprivation can cause neuronal demise [2].

Mitochondria not only provide energy to cells, but they also play an important role in cell signaling that is critical for cellular function. They can directly dictate cell survival by controlling physiological processes such as calcium homeostasis, cell proliferation, differentiation, cell cycle, protein synthesis, amino acids metabolism, and apoptosis signaling [3, 4]. Mitochondria, along with producing ATP, conduct critical metabolic activities and are decisive of cell death and survival. They serve as a nexus for apoptosis signals generated together by external and internal signals. As a result, the mitochondria occupy dominant importance in the cellular organelle hierarchy, allowing them to either encourage or conclude the cell's healthy life [5-7]. Mitochondria are required for neuronal activity due to neurons' inadequate glycolytic capability, which renders them extremely reliant on aerobic oxidative phosphorylation (OXPHOS) for energy [2, 8, 9]. OXPHOS, on the other hand, is a key source of hazardous endogenous free radicals such as hydrogen peroxide (H_2_O_2_), hydroxyl radical (**^.^**OH), and superoxide (O_2_**^.-^**) radical, which are all produced during regular cell respiration. When the electron transport chain (ETC) is blocked, electrons pile up in complex I and coenzyme Q, where they are transferred effectively to molecular oxygen to produce O_2_**^.-^**, which can then be detoxified by mitochondrial manganese superoxide dismutase (MnSOD) to produce H_2_O_2_, which in turn is transformed to H_2_O by glutathione peroxidase (GPx). Nevertheless, O_2_**^.-^** in the company of nitric oxide (NO**^.^**) produced by nitric oxide synthase (NOS) during the conversion of arginine to citrulline can result in the creation of peroxynitrite (ONOO**^-^**). Moreover, Fenton and/or Haber–Weiss reactions can turn H_2_O_2_ hazardous **^.^**OH in the vicinity of reduced transition metals. The accumulation of potentially harmful quantities of reactive oxygen species (ROS) is an outcome of normal mitochondrial respiration and homeostasis [10]. ROS and reactive nitrogen species (RNS) are well understood to serve a dual role as they can be useful as well as destructive to biological systems [11]. Oxidative stress arises when the level of free radical species generated exceeds the cells' ability to counteract them, resulting in mitochondrial malfunction and neurological damage. Mitochondrial reactive species have a variety of cellular targets, such as mitochondrial elements (lipids, proteins, and DNA). Because mitochondrial DNA (mtDNA) lacks histones and has a limited ability for DNA repair, it is an extremely sensitive target for oxidative stress [12]. Several recent reports have suggested that mitochondrially generated ROS may have a role in the initiation and advancement of neurodegenerative disorders (NDDs) in the elderly population [13-18]. The emerging recognition that mitochondria are at the crossroads of a cell's life and death, as evidenced by the role of mitochondrial injury in a variety of diseases, has made mitochondria an attractive choice for drug development. The intimate link between mitochondrial dysfunction and neurodegeneration has been discussed in this review. Moreover, we have explored some mitochondria-targeted therapies namely redox therapy, mitochondrial gene therapy, triphenylphosphonium (TPP) cation-based antioxidants, MitoQ, MitoVitE, MitoPBN, dimebon, *etc*.

## 2 MITOCHONDRIAL BIOGENESIS

Biogenesis of the mitochondria is the course through which new mitochondria are generated from pre-existing mitochondria since they cannot be generated de novo. Mitochondrial biogenesis includes the synthesis of mitochondrial membranes, the synthesis of mitochondrial proteins, the import of nucleus-encoded proteins, and mtDNA replication [19, 20]. In the brain, mitochondrial biogenesis takes place in the soma of the neurons, and it is regulated and controlled by multiple nuclear-encoded proteins such as peroxisome proliferator-activated receptor gamma (PPARγ) coactivator-1 alpha (PGC1α), which is believed to be the key regulator of the process. The interaction between PGC1α and nuclear respiratory factor 1 and 2 (NRF1 and NRF2) activates and overexpresses NRF1 and NRF2 leading to the activation of mitochondrial transcription factor A (Tfam), which is a critical protein involved in the transcription of mitochondrial ETC component genes by adhering to their promoter regions [21].

## 3 MITOCHONDRIA IN NEURAL CELLS: BIOENERGETICS AND DYNAMICS

Mitochondria are the home of major enzymes responsible for ATP production through the oxidation of sugars, fats, and proteins. Cells require a substantial quantity of ATP to perform their biological function. The balance between ATP supply/demand is linked to mitochondrial dynamics through fission and fusion [22].

**Fig. (1):**
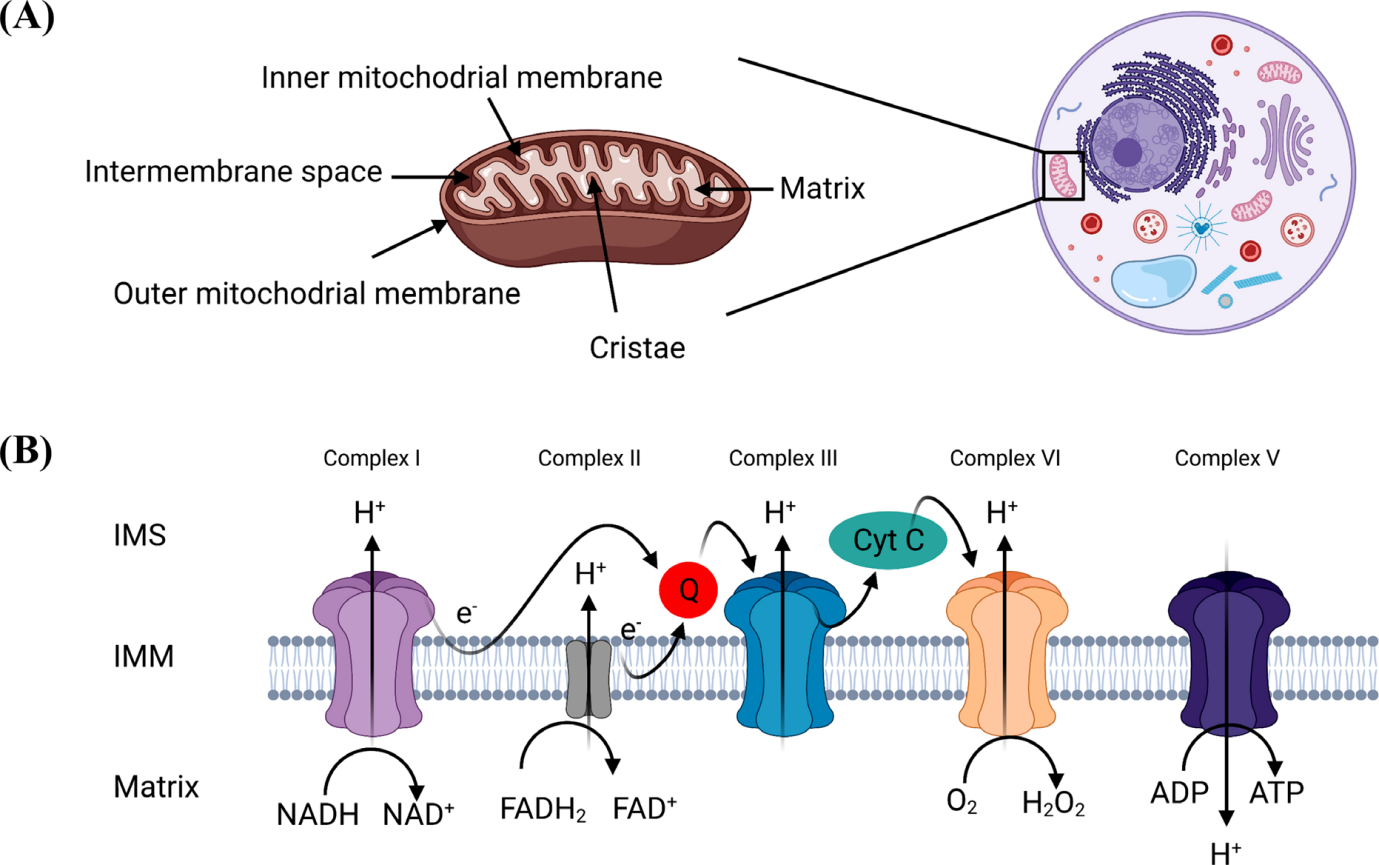
Structure and OXPHOS of the mitochondria. (**A**) Mitochondria is a cellular organelle that play role in multiple cellular processes. Structurally, mitochondria have two membranes the outer and inner membrane. Both membranes are separated by an intermembrane space. The outer membrane separates the mitochondria from the cell cytoplasm while the inner membrane separates the matrix from intermembrane space. Inner membrane is differentiated and extend into the matrix forming cristae. (**B**) Membrane bound electron transport chain in addition to complex V are responsible for ATP production through OXPHOS. The process involves electron flow from complex I, complex II, complex III, complex VI, and complex V. Created with BioRender.com.

Because of the nature of the metabolic activity of the brain, neurons consume around 20% of body glucose, which needs nonstop delivery of oxygen and nutrients through blood circulation [23, 24]. Once glucose is uptaken by the cell, it is metabolized into pyruvate molecules that are metabolized with oxygen *via* mitochondria tricarboxylic acid (TCA) cycle to feed the bioenergetic engine. Mitochondrial bioenergetic machinery contains transmembrane respiratory chain complexes protein complexes (I-IV) forming the ETC. Moreover, the proper assembly of these complexes with F_1_F_0_ATP synthase (known also as complex V) is necessary for ATP production throughout OXPHOS (Fig. **1**) [25, 26].

**Fig. (2):**
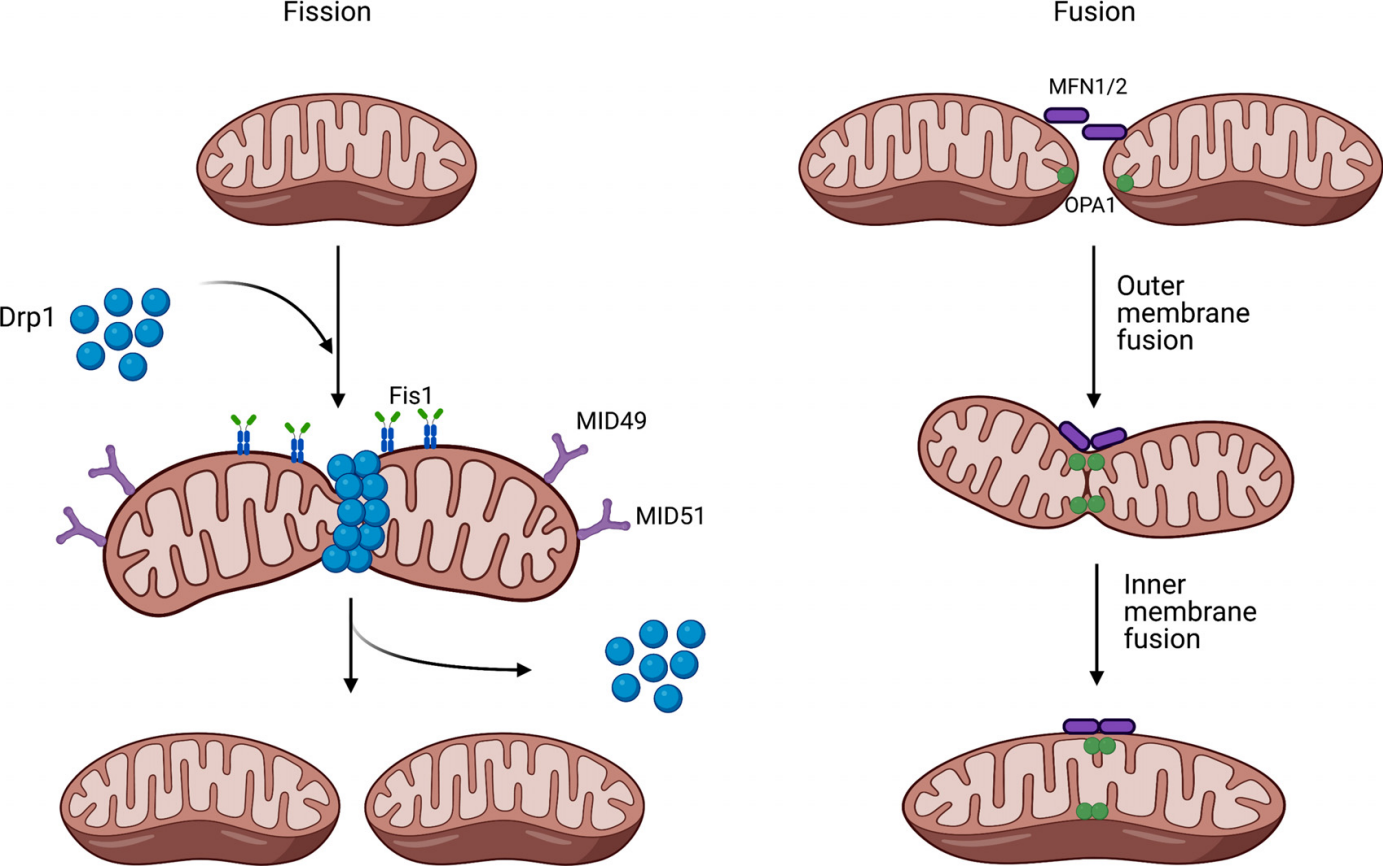
Mitochondrial dynamics. Mitochondria are very dynamic organelle that go through fission and fusion. Fission or mitochondria division can occur once Drp1 forms a ring around the mitochondria once recruited by Fis1, MID49, and MID51. Fusion or fuse of two mitochondria together occurs once multiple OMM proteins MFN1/2 and OPA1 are recruited to the surface of the mitochondria. Created with BioRender.com.

Mitochondria are extremely dynamic cellular components, which undergo constant remodeling by altering their size and number *via* two opposing controlled processes: fusion and fission, determined by dynamin-related GTPases (Fig. **2**) [27]. Mitochondrial fusion is regulated by optic atrophy protein 1 (OPA1) and mitofusins 1 and 2 (MFN1 and MFN2). There are two forms of OPA1, the long isoform that anchors to the inner membrane and the short and soluble isoform that maintains the shape of the cristae [28]. Mitochondrial outer membrane GTPase, MFN1, and MFN2, facilitate the tethering of the two neighboring mitochondria by forming homo and heteroligomeric complexes [29, 30]. While MFNs are important for outer mitochondrial membrane fusion, the GTPase OPA1 is critical for inner mitochondrial membrane fusion. Evidence also proposed that OPA1 has a function in maintaining the shape of the cristae and the loss or mutation in OPA1 may result in an alteration in mitochondrial network morphology [31]. Interestingly, OPA1 requires only one (MFN1) of the two mitofusins to function normally [32].

Mitochondrial fission depends mainly on a large family of dynamin-like GTPase including cytosolic dynamin-related protein (Drp1). Drp1 may self-assemble into multimeric spiral-like structures on the outer mitochondrial membrane, which are essential for mitochondrial fission to occur [33]. Overexpression of Drp1 was found to cause fragmentation of the mitochondria, while dominant-negative mutation was linked to aberrant brain development because of a defect in fission in the mitochondria and the peroxisomes [33-35]. In addition to Drp1, fission is promoted by several other proteins that work on the recruitment of Drp1, such as the mitochondrial fission protein 1 (Fis1), mitochondrial fission factor (Mff), and mitochondrial dynamics proteins of 49 and 51 kDa (MiD49 and MiD51, respectively) [36, 37].

Because healthy mitochondria are critical for cellular survival, even little changes in mitochondrial homeostasis can have a major influence on the cell's function and integrity. Proteinopathies is the formation of misfolded and unfolded proteins that has a great impact on cell survival, which was found in many neurodegenerative diseases [38]. The existence of a quality control machinery is critical to overcome any change in mitochondrial homeostasis, which works on different levels: molecular, organellar, and cellular. If mitochondrial function declines, molecular chaperons are activated by mitochondrial unfolded protein response (UPR) that promotes repair and recovery of the mitochondrial network and maintains normal cellular function [39, 40]. In response to UPR, damaged proteins are refolded or removed from the mitochondria [39]. Despite the capacity of chaperons to restore protein folding equilibrium, cells poorly adapt to prolong UPR since the cell becomes in a persistent mitochondria recovery leading to an increase in the accumulation of damaged mtDNA, which significantly contributes to aging-associated neurodegenerative disorders [41-45]. In these conditions, the change in intracellular nutrients, functional mitochondria, and ROS level that compromises the integrity of the proteome is influenced by vitagenes that encode for heat shock proteins (Hsp), thioredoxin, thioredoxin reductase, heme oxygenase 1, and sirtunin, all of which can be upregulated by Nuclear erythroid 2-related factor 2 (Nrf2) [45-47].

## 4 MITOCHONDRIAL PATHOLOGY IN NDDs

### 4.1 Mitochondrial Respiratory Complex Defects

Defects in OXPHOS complexes may cause disturbance in the electron passage and proton pumping through the complexes, resulting in decreased mitochondrial function and hence reduced ATP synthesis [48-50]. First mitochondrial dysfunction was discovered in Sweden in 1962 when Rolf Luft of the University of Stockholm studied a case of a woman with severe fatigue and muscle weakness with significantly elevated body temperature. Generally, when the ADP amount is low, mitochondrial substrates are not oxidized. However, in the case of this woman, mitochondria were overactive, generating heat rather than cellular energy despite the low ADP level [51]. Since Rolf Luft`s report, various diseases have been linked to mitochondrial defects, which are mainly affecting muscle [52] and brain [53, 54] tissues, both of which require a huge amount of ATP.

Altered expression of encoded OXPHOS complexes subunits genes of mtDNA and nuclear DNA contribute to the oxidation metabolism defects in several diseases, including Alzheimer’s disease (AD) and schizophrenia [55]. Downregulation of mtDNA genes of complex I subunits, such as ND4 and ND15, was detected in the temporal cortex [56] and a decrease in its enzyme activity in AD patients [57, 58]. Differential expression was also detected in complex III and IV in the hippocampus and inferior parietal lobule of AD patients [55, 59].

According to several investigations, there is a link between the inhibition of the activity of complex I and neuronal cell apoptosis [60] after generating mouse models with specific complex I activity inhibition [61-63]. These models have been generated by using pharmacological inhibitors of complex I; MPTP (1-methyl-4-phenyl-1,2,3,6-tetrahydropyridine), rotenone, or Annonaceous acetogenins [64-66]. Using MPTP caused deterioration of dopaminergic neurons of the substantia nigra, which is a manifestation of Parkinson’s disease (PD) [67]. These models showed a significant decrease for different mtDNA gens, including complex 1 and ATP synthase subunits [68].

In addition to the defect in ATP production, ROS production can be increased *via* scaping protons from defective complex I and III as they are the primary sources of ROS in both physiological and pathological conditions [69]. Changing the expression of subunit genes of OXPHOS can alter the efficiency of the ETC in eliminating the excess of ROS, and subsequently resulting in the accumulation of ROS [70] owing to brain functional changes during aging and defects in the mitochondrial respiration chain, in which complexes I, III, and IV appear to be the most affected [71]. In skin fibroblasts, an anticorrelation was reported between the severity of complex I assembly and enzyme defects and increased ROS production [72, 73]. In addition, it was found that complex III dysfunction was also associated with ROS production in isolated lymphocytes’ mitochondria from complex III deficiency patients [74]. Another study was conducted on six complex III-deficient patients with BCS1L mutations [75]. It was found that superoxide production was increased as OXPHOS complexes I, III, and IV defects severity raised in parallel with decreased production of antioxidants [38].

### 4.2 Decreased Mitochondrial Free Radical Clearing Ability

A minimal amount of ROS is vital for different physiological activities [76]. It is involved in the maintenance of essential neural progenitors [40], redox signaling [41], and the immune system by directly killing pathogens [76]. However, an imbalance between overproduction and insufficient clearance of ROS [77, 78] has been shown to cause NDDs such as AD [79] and PD [80]. Overproduction of ROS can directly damage DNA, proteins, and lipids and subsequently impairs mitochondrial functions [78]. Therefore, the antioxidative defense mechanism by which mitochondria clear ROS and protect its component is very efficient and regulated.

The antioxidative defense mechanism exerts its function in three different pathways, all of which are executed in the mitochondria. The first pathway involves superoxide dismutase (SOD)-2 and catalase, which are capable of neutralizing ROS activity. The second pathway involves peroxiredoxins 3 and 5 (Prx3 and Prx5) enzymes. They rely for their regeneration on thioredoxin (Trx) and thioredoxin reductase (TRx2). The third pathway involves GPx1, GPx4 and glutaredoxins, which rely on GSH and glutathione reductase (GR) to renew GSH [81]. There are other antioxidants, which help in ROS clearance, such as NADPH, which depends on four enzymes located in the mitochondrial matrix for its regeneration [82] on intermembrane cytochrome c that removes superoxide to make it available for oxidative phosphorylation of ADP [83].

Defects of mitochondrial complexes and mitochondrial ROS clearance ability have been documented in NDDs. Mutations of complex I subunits are correlated with 40% of all mitochondrial defects [84]. In a normal state, dopamine is oxidized by monoamine oxidase to produce hydrogen peroxidase [85]. However, in a state of ROS overproduction, dopamine is non-enzymatically oxidized by superoxide, resulting in the generation of toxic oxidants [86]. Mutations in complex II subunits and their correlation with ROS production have also been documented. Qp of complex II is a vital site for electron transfer between ubiquinone and ubisemiquinone radical. Mutations in the district Qp site were shown to be involved in ubisemiquinone destabilization, resulting in scaping an electron to interact with false acceptors such as molecular oxygen, producing highly reactive ROS [87]. Dysfunction of brain mitochondrial complex II is a characteristic of Huntington's disease (HD) and different NDDs. It was shown that inhibiting the activity of complex II by using Nitropropionic acid can mimic HD-like pathology and symptoms [88] and increase the production of ROS in neurons [89].

The ROS clearance ability of mitochondria has been compromised in many NDDs. In AD, the defects are thought to be caused by a combination of high iron levels, low GSH levels, and mitochondrial complex I defect [90]. MPP+ treatment of dopaminergic PC12 cells reduced the expression of both antioxidant enzymes Trx1, Trx2, and Trx5 [91]. In addition, the aggregation of amyloid-β-peptide (Aβ) is correlated with mitochondrial antioxidant system defects in AD [92]. Aβ is responsible for H_2_O_2_ accumulation and contribution to mitochondrial defects [93]. It was found that SOD2 downregulation was correlated with the accumulation of brain Aβ levels in human amyloid precursor protein (hAPP) transgenic mice [94]. Other NDDs have also shown a decrease in Prx3 expressions, such as post-mortem brains of Down syndrome (DS) patients [95, 96] and in the motor neurons of familial amyotrophic lateral sclerosis (ALS) patients [97]. Selective mGSH depletion accelerates the onset of Huntington's disease (HD) symptoms in mice after *in vivo* injection of 3-nitropropionic acid [98].

The above discussed findings show a possible important role of mitochondrial antioxidant ability in the prevention of NDDs. Hence, boosting the antioxidant ability could be a potential preventive or therapeutic approach in the future.

### 4.3 mtDNA Lesions

MtDNA, circular DNA, can make its own RNAs and proteins since it has its own genetic material and machinery. MtDNA encodes 13 mitochondria-associated polypeptides, two rRNAs, and 22 tRNAs that are involved in mitochondrial protein synthesis [99, 100]. Even though mtDNA is highly protected by the antioxidants from the low level of OS, it is still sensitive to OS-induced mutations because of the low efficiency of mtDNA repair enzymes, and the physical closeness of mtDNA to free radical formation hotspots [101]. It has been shown that mutations of mtDNA resulted in insufficiency of mitochondrial complex activities, resulting in mitochondrial OXPHOS defects, ROS overproduction, and subsequently, cell apoptosis [102, 103] as observed in aging and a wide variety of NDDs, such as AD, PD, HD and ALS [104-107].

Genetic studies revealed mtDNA mutations in PD and AD to be associated with mitochondrial-specific OXPHOS complexes defects [108]. For example, specific knockout in mtDNA resulted in complex I deficiency of PD- affected neurons [109, 110]. In AD brains, it was found that cytochrome *c* oxidase-deficient neurons obtain a greater level of mtDNA mutations compared with age-matched controls [111]. In ALS, mutant SOD1 was found to significantly reduce voltage-dependent anion-selective channel protein 1 (VDAC1) activity, resulting in a significant decrease in energy production in mitochondria [112, 113]. Likewise, in transgenic ALS mice’ brain, levels of mtDNA damage were found to be 30-fold higher in the motor cortex as compared to spinal motor neurons [114, 115].

### 4.4 Mitochondrial Calcium Dyshomeostasis and Mitochondrial Permeability Transition Pore (mPTP)

Calcium signalling is involved in a variety of physiological processes, including muscle contraction, neuron excitability, and cell migration [116, 117]. The main storage sites of calcium are ER and mitochondria. Not surprisingly, therefore, mitochondria are essentially involved in calcium homeostasis maintenance through calcium buffering, which keeps calcium levels between 50 and 500 nM in numerous types of normal cells, and its interactions with other channels or organelles, such as ER [118-120]. Dysregulation of calcium may lead to defects in mitochondrial dynamics, function, and metabolism [121, 122].

Disturbance of calcium buffering capability of mitochondria results in an overload of calcium, which is one of the main features of mitochondrial abnormalities in NDDs. In AD, PD, HD, and ALS patients, calcium overload has been detected in affected regions, and similar results were obtained in the animal and/or cellular models of these diseases [123-126]. Calcium overload was found to cause ROS overproduction and activation of mPTP formation to enhance calcium efflux and ROS accumulation. Consequently, mitochondrial respiration complexes are damaged, cytoplasm is flooded with pro-apoptotic chemicals, mitochondria are swelled up and its membrane gets ruptured [127-129]. The main components of mPTP are voltage-dependent anion channel (VDAC), adenine nucleotide translocase (ANT), and cyclophilin D (CypD), which are embedded in the outer mitochondrial membrane (OMM), inner mitochondrial membrane (IMM), and matrix, respectively [130]. Translocation of CypD to IMM for binding with ANT is the initial step of the formation of mPTP [131-133]. The formation of mPTP in neurons causes apoptosis and cell death [138], which is widely detected in affected regions of different NDDs. CypD levels were found to be considerably higher in AD-affected areas, temporal pole, and hippocampi. Likewise, CypD was overexpressed in the brains of transgenic AD mice (including hippocampus and cortex) as well aged mice [127, 134]. Another study screened the expression CypD in different brain regions of rats with different NDDs. It was reported to exhibit an increase in the CypD expression levels in the disease-affected brain regions compared to normal healthy rats. This finding suggested the possible involvement of CypD in the etiology of different NDDs [135].

### 4.5 Mitochondrial Impairments in Brain Aging: Insight into the Role of Estrogen

Neurodegeneration is a progressive disorder involving brain aging. This is primarily characterized by a cognitive decline as well as a decline in physical functioning. The reason for this decline is not well understood. However, several hypotheses, causes, and factors have been proposed to explain the deterioration in cognitive and physical function [136]. One potential cause is the poor production of mitochondrial proteins due to the impairment of cellular energy production [137]. A link between the deterioration in mitochondrial function and aging has been suggested [138]. Moreover, mitochondrial impairment is believed to be one of the main reasons for neuronal cell death [139, 140]. Multiple shreds of evidence also revealed that NDDs may be caused by mitochondrial dysfunction [141]. In the past few years, studies with a focus on age-related NDDs such as AD, PD, and HD have established the link between mitochondrial dysfunctions and NDDs [142-145]. Several *in vivo* investigations have found that mitochondrial function in the brains of AD patients is impaired [146, 147]. Aging was found to cause a decline in mitophagy, which functions to remove defective proteins including dysfunctional mitochondria. The reduction of mitophagy may lead to deterioration in mitochondrial function because of the accumulation of defective proteins, and mutations along with oxidative injury. As a result, mtDNA volume, integrity, and functionality are reduced, which impairs mitochondria and may manifest decreased oxidative capacity and ATP production along with a substantial rise in ROS production [148]. Moreover, recent studies have described that mitochondria serve as targets for estrogen effect as well as essential intermediaries of steroid hormones’ biogenesis including estrogen [149]. Estrogen regulates mitochondrial structure and function by increasing the expression of respiratory complexes, antioxidant particles, and anti-apoptotic factors [150]. Predominantly, estrogen is produced in the ovaries and adrenal glands, but it is also produced by several tissues including adipose, breast stromal, and brain tissues. Estrogens exert their effects by acting on estrogen receptors α and β (ERα and ERβ), along with G-protein coupled estrogen receptor 1 (GPER1 also known as GPR30) [151]. ERα and ERβ are considered to be transcription regulators which influence gene transcription by binding to genomic and mtDNA. Additionally, ERα and ERβ initiate intracellular signaling pathways leading to the modification of transcriptional reactions, involving mitochondrial structure and function by interacting with plasma membrane-associated signaling proteins. Estrogens have different forms such as estriol and estradiol, which is the main female sex hormone that is engaged in mitochondrial function control [150]. Estradiol acts *via* the activation of the transcription factor NRF-1 that reacts with PGC-1α to regulate mitochondrial genes. Estrogen shows neuroprotective, neurotrophic, and antioxidant effects in the brain [152]. Several studies have indicated that during aging in women, estrogen production is decreased, thus precipitating the susceptibility of women to brain degeneration and aging diseases. The decrease in estrogen production was also correlated with significant impairment in brain mitochondrial function [153]. Aging is associated with not only sex steroid deficiency but also an augmentation in free radical generation causing damage to mitochondria. Impaired mitochondria have additional susceptibility to generate more free radicals that can damage cellular function including estrogen biogenesis [138]. Hence, mitochondrial therapeutics and the improvement of mitochondrial function using estrogen may be potential future targets and/or tools to fight several critical NDDs.

## 5 MITOCHONDRIAL MEDICINE FOR NEUROLOGICAL DISORDERS

### 5.1 Mitochondria-based Interventional Medicine

Mitochondrial dysfunction has a role in the onset of a plethora of diseases. Therefore, mitochondria are currently considered a prominent pharmacological target. A vast number of experiments have confirmed the beneficial outcome of mitochondria-targeting compounds (MTCs) in the management of various neurological disorders such as AD, PD, HD, and MS [154-157]. Although no criteria exist to characterize MTCs, Zinovkin *et al.* proposed that for any chemical to be classified as MTC 90% of the total quantity injected into the cells should get accumulated in the mitochondria [158]. In 1995, Thiobutyl-TPP bromide was identified as the first mitochondrial targeted compound by Murphy *et al.* [159]. Since then, dozens of MTCs have been involved in *in vitro* and *in vivo* experiments.

In contrast to other organelles inside the cells, the mitochondria have some distinctive features that can be utilized to synthesize/identify a MTC compound. For example, the mitochondria have negative charges in their cores and high transmembrane potential between the matrix and the intermembrane space (average value of 180 mV) [160]. The mitochondrial membrane potential is physiologically crucial to generate ATP molecules. However, it can be utilized to target the mitochondria *via* the utilization of cations, which will be directed to the negatively charged part of the mitochondria (the matrix) [161]. Another mitochondrial unique feature is the presence of phospholipid cardiolipin in their inner membrane. This phospholipid reinforces respiratory chain complexes but it can also be used to deliver compounds into the mitochondria [162]. Additionally, the mitochondria possess a specialized protein import system that binds to specific amino acid sequences [163]. This system can be exploited to deliver compounds inside the mitochondria.

Once inside the mitochondria, possible effects of MTCs include the decoupling of the mitochondrial membrane potential and its usage for mitochondria-dependent ATP generation (also called mitochondrial uncoupling), the induction of mitochondria-dependent programmed cell death, and the reduction of ROS level. Moreover, MTCs can be designed as a sensor to detect ROS level. MTCs may exert one or a combination of these effects. MTCs often have other concomitant effects because of the complex mitochondrial processes. Specific MTCs can lower ROS levels while acting as a mitochondrial uncoupler at certain doses [164]. Other MTCs can even induce ROS production and decreased ATP levels when used at high concentrations [165, 166]. Therefore, extensive *in vivo/in vitro* research is required to investigate the effect of any newly identified MTC.

### 5.2 Redox Therapy

A redox reaction is a chemical process that involves the movement of electrons between two molecules. In an oxidation-reduction redox reaction, the oxidation number of a molecule varies by acquiring or releasing an electron. While molecular oxygen (O_2_) is essential for life, its univalent reduction within the body causes the generation of various ROS species. Cells can clear ROS and protect themselves from their deleterious effects *via* the use of antioxidant compounds [167]. These antioxidants are either from endogenous or exogenous sources. Antioxidants include enzymes such as SOD, catalase, and GPx/GR, minerals such as copper and zinc, vitamins such as vitamin C and E, and other chemicals such as bilirubin and uric acid [168]. Lipid-soluble antioxidants like carotenoids, quinones, and certain polyphenols, as well as water-soluble antioxidants like ascorbic acid, are all examples of dietary antioxidants [168].

The balance between the rate of ROS generation and clearance is critical for OS. If the generation of ROS increases above the capacity of endogenous antioxidants or if endogenous free radical clearing ability was diminished, nutritional and exogenous sources of antioxidants might play a significant role to maintain redox balance and decrease stress levels. In fact, the beneficial effect of antioxidants has been reported in various *in vitro/in vivo* research (Table **1**). Therefore, redox therapy is a hopeful approach to treating NDDs.

There has been a tremendous effort to identify nutraceutical antioxidants as novel therapies for neurodegenerative diseases. One of the natural product families that showed promising results in various studies is plant polyphenols. This family includes a number of flavonoids and non-flavonoids compounds, which differs according to the number of hydroxyl groups, and the presence of other substituents [169]. The list of the studied natural phenols includes curcumin, epigallocatechin-3-gallate (EGCG) (the flavanol found in green tea) and resveratrol (a stilbene found in grapes and in red wine) [170].

The beneficial effect of plant polyphenols has been an emerging research focus in many neurological diseases and has been more evident in conditions where oxidative stress is implicated in the pathophysiology such as AD and PD [171]. The antioxidant properties of polyphenols, are demonstrated by their ability to scavenge reactive radicals. Some polyphenols neutralize ROS by trapping chain-propagating free radicals, either *via* hydrogen atoms transfer or electron transfer [172]. Other polyphenols prevent the deleterious effect of ROS on a substrate *via* the inhibition of oxidation promoters (such as metal ions, dioxygen and pro-oxidative enzymes), thus, reducing their redox potentials [172]. Nonetheless, the exact mechanism/s of action of polyphenols is/are not fully understood. Studies suggested that polyphenols might have multiple mechanisms of action and that they are capable of modifying gene expression, miRNA and proteins [173]. In line with that, emerging evidence shows that the polyphenols – mediated neuroprotection is likely achieved *via* the activation of vitagene signaling pathways [171].

The term “vitagenes” is referred to a group of genes involved in preserving cellular homeostasis during either physiological or pathological stressful conditions [174]. Research found that the expression of numerous vitagenes decreases with age. Specific supplement intake positively affects vitagenes expression and ameliorates the unwanted effect of aging [174]. Notably, the number of genes that may be classified as vitagenes rises if aging was associated with disease [174]. Some important vitagenes are listed in Table **2**. Notably, they are all directly or indirectly linked to the mitochondria.

Research showed that polyphenols initiate phase 2 response, leading to the expression of various Nrf2-dependent antioxidant vitagenes, including the aforementioned Hsp70, HO-1, sirtuins system and many others [175]. Nrf2 is a key transcription factor that governs hundreds of cytoprotective genes. Notably, Nrf2 activation induces a mild stress response, which promotes cell survival and induces a healthy physiological steady state. However, prolonged Nrf2 activation may results in an adverse outcome, indicating that Nrf2 has a hormetic-like behavior [176, 177]. Hormesis has emerged as a central concept in biological and biomedical sciences. Hermetic dose-response may be established when a low certain dose leads to stimulation while a high dose of the same compounds leads to inhibition, that is, a biphasic dose-response relationship [178]. The principle of hormesis appears to be applicable to the downstream target of the Nrf2 pathway as well. Research showed that the antioxidant effect of HO-1 against oxidative and nitrosative stress is abolished upon excessive upregulation of HO-1 system [179-181]. A possible explanation is the accumulation of its by-products such as carbon monoxide, iron, and bilirubin [181].

An increasing number of studies support the usefulness of polyphenols, more evidently when used with other drugs [182]. Although some conflicting results have been reported, these could be attributed to various factors such as different experimental settings and clinical conditions. More work is needed to investigate these findings. However, the overall current body of the data strongly suggests the nutraceutical value of plant polyphenols.

### 5.3 Mitochondrial Permeability Transition Inhibition

Since the discovery of cyclosporin A (CsA) as the first inhibitor of mPTP four decades ago [183], cumulative evidence showed that mitochondrial dysfunction and mPTP opening is considered the primary mechanism of apoptosis in many NDDs [184]. Consequently, several CsA- related mPTP inhibitors have been identified and tested. Although these inhibitors showed promising results in preclinical research, we are still far from a complete understanding of their molecular mechanism. This is mainly attributed to the polypharmacology of CsA and its derivatives [184]. In addition, the process of mPTP opening itself is not completely understood. Various proteins such as IMM proteins: ANT and mitochondrial phosphate carrier (PiC), and OMM proteins: VDAC and BCL-2 family members BAK/BAX; have been suggested as key players in mPTP creation. Nonetheless, almost all of these candidate proteins did not show consistent results in knockout/over-expression genetic studies [184].

To date, the only protein that has consistently proved to serve a direct function in regulating mPTP opening is peptidyl prolyl cis-trans isomerase (PPIase), also known as CypD [185]. CypD possesses a Peptidyl-Proline Isomerases activity, which is crucial for mediating pore opening. Research showed that CypD inhibition decreases the susceptibility of the cells to Ca^2+^ and ROS *via* the inhibition of mPTP formation [186]. However, if Ca^2+^ concentration increases above a certain level, mPTP formation occurs regardless of CypD inhibition [186]. This suggests that CypD plays a regular role and is not structurally involved in the process. The scientists proposed that the mPTP formation process starts upon the binding of ANT to CypD in IMM. This binding along with the help of other proteins consequently mediate the formation of tunnel-like structures across both IMM and OMM [187].

The vast majority of known mPTP inhibitors are CypD-dependent as they target CypD specifically [188]. CsA is the most tested and best characterized CypD-dependant mPTP inhibitor. In mAPP mice models of AD (mice expressing a mutant form of hAPP), CsA treatment protected the neurons from oxidative damage *via* reducing the generation rate of ROS [189]. Moreover, CsA treatment attenuated Ca^2+^ imbalances and mitochondrial swelling [189]. CsA-treated mice had substantially improved scores in learning and memory tests [189]. Similar findings were observed in CypD-deficient mAPP mouse model [189]. The beneficial effect of CypD inhibition was also demonstrated in PD-related models. CypD-deficient mice treated with mPTP showed significant cytoprotection compared with control mice [190]. The isolated mitochondria of CypD-deficient mice demonstrated higher resistance to MPP+ treatment and lower ROS level in comparison to the wild type [190]. Interestingly, the expression of apoptotic markers in CypD-deficient mice was not increased, and the beneficial effect of CypD-deficiency was apparent only with an acute regimen of mPTP treatment [190].

Unfortunately, the beneficial effect of CsA mediated mPTP inhibition was difficult to be demonstrated clinically due to certain limitations like toxicity, inhibitory effect on the immune system, and limited bioavailability in the central nervous system (CNS) [191]. Therefore, researchers are currently working to synthesize and identify alternatives to CsA. One of the approaches used to identify new mPTP inhibitors includes virtual screening to identify the best fit synthetic, and semisynthetic molecules. In this approach, the qualified molecules can be further modified into novel more active analogs with the aid of modeling techniques such as *in-silico* QSAR modeling and molecular docking. For example, Valasani *et al.* used diastereomeric crystallization and pharmacophore modeling to generate multiple selective CypD inhibitors [192]. Likewise, Belkacem *et al.* synthesized a group of nonpeptidic cyclophilin inhibitors that are structurally different from CsA, and possess PPIase inhibitory activity [193]. In addition, it has been reported that some quinoxaline derivatives such as quinazoline urea analogs bind and inhibit CypD, and demonstrated strong inhibitory ability against Ca^2+^-dependent rat liver mitochondrial swelling [194]. These compounds are promising CsA alternatives if they prove to be safe clinically.

### 5.4 Mitochondrial Gene Therapy

Gene therapy for NDDs has progressed significantly over the past years. This is attributed not only to the discoveries related to the role of genetics in the etiology of these disorders but also to the advance in the technology that delivers the therapeutic DNA/RNA segments to the desired tissue. Gene therapy may result in gene silencing to control the gain of function mutations or gene overexpression to compensate for the loss of function mutations. Gene therapy has many advantages in comparison to traditional medical treatment. It has a more permanent effect and does not require repetitive doses. Gene therapy can also treat tissue that has been consistently unresponsive to medical treatment.

There are special viral/non-viral vectors that have been used to carry the transgene to their targets in the CNS. These transgenes might carry codes to express therapeutic proteins, cDNA for gene addition, Cas9/gRNA for gene editing, small interfering RNA (siRNA) *etc*. [195]. The most commonly used vector in NDDs is the Adeno-associated viruses (AAV) [196]. Dozens of AAV, which are classified in 13 serotypes, have been identified [197]. AAV2 in particular is commonly used clinically for gene therapy of NDDs as it is relatively safe and has consistent expression in neurons [197]. For example, AAV2-NGF (nerve growth factor) gene therapy *via* basal forebrain injection proved to be well tolerated in AD patients [198]. AAV2-NGF carries the codes for the NGF), which is an endogenous neurotrophic-factor protein that can protect degenerating cholinergic neurons. AAV2-NGF was used in the multicenter randomized clinical trial as a treatment for AD [198]. However, there was no improvement in cognition after 24 months of treatment [198].

Since mitochondrial dysfunction plays a significant role in the pathology of NDDs, mitochondrial gene therapy might have great potential as a treatment strategy. Gene therapy that mediates the productions of various regulatory factors of mitochondrial ROS and mitochondrial dynamics was able to protect neurons *in vitro* in PD and AD experimental models [199, 200]. However, clinical studies did not show promising results [197]. This could be because the patients in these studies were too mature for treatment because they had already experienced substantial neurodegeneration. Nonetheless, scientists still believe that mitochondria-targeting gene therapy for NDDs is a promising area of research, particularly for NDDs with strong evidence of mitochondrial dysfunctions such as PD.

### 5.5 TPP Cation-based Antioxidants

As mitochondria are the main course of ROS in the cells, antioxidant drug that targets ROS production needs to be accumulated in the mitochondria to mediate its effect. This could be achieved *via* the conjugation of the antioxidant molecule to a mitochondria-targeted peptide [201] which is an N-terminal mitochondrial targeting signal (MTS) peptide that builds amphipathic helical structures with positively charged residues. The positive charge allows electrostatic interactions with the negatively charged mitochondrial interface [201]. Subsequently, the molecule is transported inside the mitochondria. Alternatively, the antioxidant can be conjugated to a lipophilic cation that can diffuse through the phospholipid bilayer of the mitochondrial membrane [202]. The charged cations are functionally arranged on the hydrophobic surface of lipophilic cation molecules. This arrangement renders the lipophilic cation capable of diffusing through the mitochondrial membrane with minimal activation energy and without the need for pores or transporter proteins [202]. The most commonly used lipophilic cation to deliver antioxidants to the inside of the mitochondria is TPP [201]. TPP contains an intermediate positive charge of phosphorus which attracts the TPP toward the negatively charged mitochondrial matrix [201]. In the coming section, we will briefly discuss some examples of TPP cation-based antioxidants.

### 5.6 MitoQ

MitoQ is a mitochondria-directed compound made up of ubiquinone molecule (the oxidized form of CoQ10) that is covalently bonded to TPP molecule [203]. Thus, MitoQ can diffuse through the mitochondrial membrane and aggregate in the mitochondrial matrix. Inside the mitochondria, MitoQ detoxifies ROS and consequently reduces lipid peroxidation and mitochondrial injury [204]. Moreover, ubiquinol (the reduced form of CoQ10) can be oxidized back to ubiquinone and used continuously by complex II of the ETC, which makes MitoQ superior to other mitochondria-targeted antioxidants [201].

Since the discovery of MitoQ in the 1990s, it has been involved in multiple studies to investigate its potential in NDDs [205]. With regards to PD, MitoQ showed promising results in *in vitro* studies. MitoQ pre-treatment prevented mitochondrial translocation of Drp1 and the consequent mitochondrial fragmentation in 6-hydroxydopamine treated SH-SY5Y cells [206]. In the same study, MitoQ also prevented the trafficking of the pro-apoptotic factor, Bax, to the mitochondria and enhanced the survival of the SH-SY5Y cells [206]. In MPTP-induced mouse models of PD, MitoQ treatment improved motor deficit and increased dopamine levels and the expression of tyrosine hydroxylase in the substantia nigra [207]. Similar beneficial effects were also observed in AD-related research [207]. MitoQ reduced both Aβ accumulation and Aβ-induced OS. In the genetic murine model of AD, namely 3xTg-AD mouse, MitoQ delayed the onset of cognitive dysfunction in Morris Water Maze tests [208]. The obtained brain samples revealed evidence of the therapeutic effect at molecular levels such as decreased caspase 3 and 7 activity, Aβ immunoreactivity, and oxidative stress markers [208].

Although MitoQ passed phase I and phase II clinical trials with success as a treatment option for patients with Hepatitis C virus [209], it failed to demonstrate clinical benefits in PD patients. One possible reason is that the degree of dopaminergic neuronal impairment in PD patients had exceeded the protective capacity of MitoQ. More study is required to confirm the clinical benefit of MitoQ in NDDs given their robust encouraging findings in *in vitro* and *in vivo* investigations.

### 5.7 MitoVitE

Vitamin E is a collection of naturally occurring plant lipids known as tocopherols and tocotrienols [210]. The members of the vitamin E family differ in their methylation pattern. However, they all possess an electrophilic hydroxyl group that can extinguish carbon radicals effectively [210]. This hydroxyl group is responsible for the powerful antioxidant effect of vitamin E [210]. Physiologically, Vitamin E is highly linked to the nervous system. In fact, the manifestations of Vitamin E deficiency are mainly neurological [211]. Interestingly, it seems that the brain is specially adapted to vitamin E usage. Vitamin E’s biological life in the brain is gradual and varies with its concentration from region to region [212, 213], which also reflects the presence of tissue-specific regulatory mechanisms for vitamin E storage and usage.

Vitamin E appears to be useful in diseases in which OS serves a significant role in the pathogenesis [214]. Previous studies showed that a low concentration of vitamin E has a neuroprotective role in the case of glutamate excitotoxicity as well [215]. A recent questionnaire-based case-control study suggested that vitamin E intake might protect against PD. This study involved 100 PD patients and an equal number of healthy controls and showed that the quantity of vitamin E in one's diet was negatively correlated with PD incidence, regardless of sex and age [216]. This is in accordance with a recent meta-analysis research that looked at the link between PD and dietary consumption of vitamin C, β carotene, and vitamin E [217]. While there was no significant link between vitamin C and carotene, nutritional consumption of vitamin E was discovered to be protective against PD. More research is needed to investigate how MitoVitE can help elderly people and people with NDDs.

### 5.8 MitoPBN

MitoPBN is a compound that consists of coenzyme Q (quinone) and phenyl tertbutylnitrone molecules attached to TPP bromide [218]. MitoPBN was synthesized to protect against ROS-induced lipid peroxidation relying on the ability of N-tert-butyl-α-phenylnitrone (PBN) to neutralize carbon-centered radicals (R^.^) and peroxyl radicals (ROO^.^) [219].

A more complex nitrenium cation (LPBNAH) (scientific nomenclature: N-[4-(octa-O-acetylactobionamidomethylene) benzylidene]-N-[1,1-dimethyl-2-(N-octanoyl) amido]-ethyl-amine N-oxide), which is a derivative of PBN, seem to be more effective due to its high stability and selectivity [220]. This compound demonstrated a neuroprotective effect against OS and cell demise in neuroblastoma cells that were exposed to Aβ, hydrogen peroxide, and 3-hydroxykynurenine [220]. Nonetheless, there are very few studies have been done to explore the effect of MitoPBN and LPBNAH in the context of NDDs. More *in vivo* and *in vitro* research is required to investigate the potential of these promising compounds.

### 5.9 Dimebon

The antihistamine medicine Dimebon was initially utilized to manage allergies in Russia in the early 1980s. In recent times, Dimebon has been suggested as a treatment for NDDs [221]. The first crucial clinical trial of Dimebon in AD revealed that it reduced the disease's clinical manifestations [222]. In this randomized, double-blind, placebo-controlled trial of 183 individuals with mild-to-moderate AD, Dimebon showed statistically significant improvements in all essential areas of the disorder including memory, thinking, activities of daily living, behavior, and overall function. People treated with Dimebon did considerably better than placebo-treated individuals in all critical metrics of the disorder after 6 months and one year of treatment [222]. Dimebon was also found to be effective in a phase II trial done by Medivation and the Huntington Study Group with HD patients (DIMOND). Although there are highly hopeful outcomes in clinical trials, but mechanisms behind Dimebon's therapeutic effects are yet unknown. Dimebon has been shown to block NMDA receptors and voltage-gated Ca^2+^ channels in the past [223-226]. Dimebon also inhibits the entry of the mPTP caused by Aβ_25–35_ and MPP^+^, according to a prior study [227]. These findings imply that Dimebon's therapeutic effects may be attributed to its capacity to maintain neuronal calcium homeostasis and mitochondrial activity. Dimebon's mechanism of action in neurological disorders is currently being investigated by researchers.

As discussed in the aforementioned sections, the specific roles of each mitochondria-directed medicine have been illustrated in Fig. (**3**).

## CONCLUSION AND FUTURE PERSPECTIVES

Numerous research reports indicate that mitochondrial abnormality and oxidative stress are important in the pathophysiology of many NDDs including AD, PD, HD and ALS. Mitochondria are the primary source of energy for brain cells to function normally. Higher production of ROS, aberrant protein-protein interactions, and decreased mitochondrial ATP synthesis have all been linked to mitochondrial abnormalities. In early-onset, inherited, and late-onset, non-inherited NDDs, increased generation of ROS with reduced mitochondrial activity has been demonstrated to harm neurons. As a result, developing strategies to combat or reduce mitochondrial abnormality could be therapeutically beneficial. Redox therapy, mitochondrial gene therapy, TPP cation-based antioxidants have been reported to be efficient in pre-clinical and clinical investigations demonstrating their potential. Furthermore, using antioxidants in combination maybe even be more efficient than using single compounds. Dimebon, a novel therapy candidate, can improve cognitive deterioration in AD and HD patients. However, broader clinical studies with a greater quantity of individuals are required to give more conclusive evidence of these compounds' therapeutic potential. Nevertheless, the molecular mechanism of Dimebon is unknown, indicating the necessity of additional investigation, especially in animal studies. Mitochondrial-targeted medicines will open long-term options for manipulating mitochondrial function, potentially protecting against NDDs.

## Figures and Tables

**Fig. (3):**
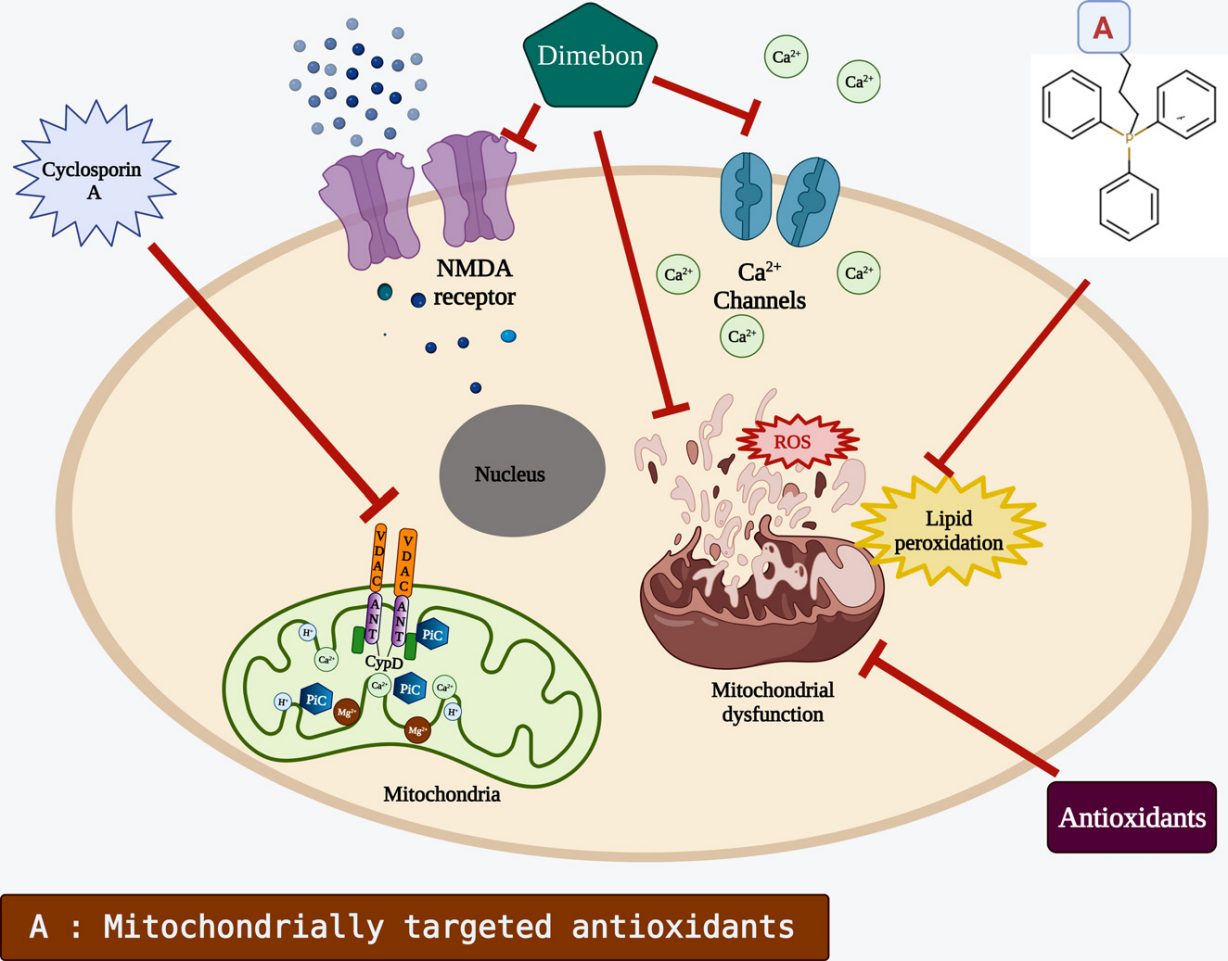
Mitochondrial medicines. Antioxidants maintain redox balance and decrease the stress level by avoiding damage of lipids, proteins, and DNA; Dimebon inhibits glutamate inflow by binding to NMDA receptors on the cell's surface. It also binds to Ca^2+^ channels, preventing a Ca^2+^ influx into the cell; Triphenylphosphonium cation-based antioxidants like MitoQ. MitoPBN *etc.* could be used to deliver antioxidants to the inside of the mitochondria; cyclosporin A (CsA) inhibits mitochondrial permeability transition pore (mPTP). Created with BioRender.com.

**Table 1:** Examples of anti-oxidant substances and their beneficial effects *in vivo* / *in vitro* in neurodegenerative diseases.

**Substance**	**Extracted ** **Compound/s**	**Clinical Beneficial Effect**	**Pass BBB**	**Disease**	**Molecular Beneficial Effect**	**References**
*Ginko biloba*	EGb761	Cognition Memory Attention	-	AD	Reduce amyloid beta aggregation and toxicity.	[228, 229]
Selenium	Selenoproteins glutathione peroxidase (GPx)	Cognitive	+	AD PD MS	Protects against amyloid beta and iron/ hydrogen peroxide-mediated neuron death.	[230, 231]
Tumeric	Curcumin	Cognition	+	AD PD	Decrease amyloid beta levels, neutralize ROS and peroxynitrite, increase GSH formation, inhibit transcription factor NF-kB*.	[232]
Cannabinoids	Dronabinol Cannabidiol	Cognition Memory Sympathomimetic effect, motor manifestation of PD Neuropathic pain	-	PD AD ALS MS	Antioxidant effect, antinflammatory effect, inhibit transcription factor NF-kB, protect dopaminergic ncurons, ncrease trophic factors, romotes, neuroglia survival, decrease demyelination.	[233-236]

**Table 2:** Some important vitagenes and their function in the cell.

**Vitagene**	**Protein**	**Function**
**BCL2**	Bcl-2	Protect against of mitochondrial-dependent apoptosis [237]
**CREB1**	CREB-1	Regulate mitochondrial synthesis [238]
**GSS**	Glutathione synthetase	Defense against reactive oxygen species [239]
**HMOX1**	Heme oxigenase-1	Regulate mitochondrial synthesis [239]
**HSP70**	Hsp70	Post translational modification of mitochondrial proteins [240]
**SIRT1 - 4**	Sirtuin-1 - 4	Post translational modification of mitochondrial proteins, regulation of mitochondrial electron transport and oxidation, Regulate mitochondrial synthesis and dynamics [241, 242]
**SOD2**	Superoxide dismutase	Defense against reactive oxygen species [243]

## LIST OF ABBREVIATIONS

AAV: Adeno-Associated Viruses
AD: Alzheimer’s Disease
ALS: Amyotrophic Lateral Sclerosis
ANT: Adenine Nucleotide Translocase
DS: Down Syndrome
ETC: Electron Transport Chain
GPx: Glutathione Peroxidase
GR: Glutathione Reductase
HD: Huntington's Disease
IMM: Inner Mitochondrial Membrane
Mff: Mitochondrial Fission Factor
MnSOD: Manganese Superoxide Dismutase
mPTP: Mitochondrial Permeability Transition Pore
MTS: Mitochondrial Targeting Signal
NDDs: Neurodegenerative Disorders
NOS: Nitric Oxide Synthase
OPA1: Optic Atrophy Protein 1
OXPHOS: Oxidative Phosphorylation
PD: Parkinson’s Disease
RNS: Reactive Nitrogen Species
ROS: Reactive Oxygen Species
siRNA: Small Interfering RNA
TCA: Tricarboxylic Acid
TPP: Triphenylphosphonium
UPR: Unfolded Protein Response
VDAC: Voltage-Dependent Anion Channel
